# Size estimation of key populations and ‘bridge populations’ based on the network scale-up method in Ukraine

**DOI:** 10.1186/s12889-024-18501-1

**Published:** 2024-04-08

**Authors:** Oksana Kovtun, Volodymyr Paniotto, Yulia Sakhno, Kostyantyn Dumchev

**Affiliations:** 1 Alliance for Public Health, Kyiv, Ukraine; 2 Kyiv International Institute of Sociology, Kyiv, Ukraine; 3 Ukrainian Institute on Public Health Policy, 24 Bulvarno-Kudryavska Street, building 3, 01054 Kyiv, Ukraine

**Keywords:** Network scale-up method, Size estimation, Key populations, Bridge populations

## Abstract

**Introduction:**

Correct estimation of the size of key and bridge populations is crucial for an efficient HIV/AIDS response in resource-limited settings, enabling efficient program planning and resource allocation. The hidden nature of these groups poses challenges to traditional methods, leading to the adoption of innovative approaches like the network scale-up method (NSUM). In this article we present the results of a NSUM study conducted in 2020 in Ukraine, focusing on four key populations and three bridge populations, highlighting challenges and contributions to development of the method.

**Methods:**

From July to September 2020, we conducted a nationally representative survey in Ukraine via computer-assisted telephone interviews, and applied the known population method and summation method to estimate social networks sizes. Results were weighted based on individual sampling probability and adjusted for social respect and visibility factors to address potential limitations.

**Results:**

Our study achieved a 20% response rate with 10,000 completed interviews. The social network size, using the known population method, was 213 people, and 125 using the summation method. Adjusting for the social respect and visibility, estimated key populations sizes were 295,857 [248,714–343,001] people who inject drugs, 152,267 [109,960–194,573] men who have sex with men, 78,385 [57,146–99,619] sex workers, and 9,963 [7,352–12,571] transgender people, detailed by age and gender. Bridge populations were estimated at 62,162 [50,445–73,879] sexual partners of people who inject drugs, 284,348 [233,113–335,583] clients of sex workers, and 13,697 [7,370–20,026] female partners of men who have sex with men.

**Conclusions:**

NSUM proves reliable for estimating key populations size with appropriate corrections. It shows promise for further use in Ukraine, considering limited geographic coverage of the integrated bio-behavioral studies to use multiplier-based methods. However, the validity concerns persist for estimating bridge populations size, emphasizing the need for further method refinement and addressing implementation issues, particularly those related to data collection.

**Supplementary Information:**

The online version contains supplementary material available at 10.1186/s12889-024-18501-1.

## Background

Accurate knowledge of key population (KP) sizes is crucial for an effective HIV/AIDS response, particularly in resource-limited settings [1, 2]. Population size estimation (PSE) plays a crucial role in planning and implementing preventive programs, developing HIV services, and allocating resources [3]. KP PSE is also necessary for estimating the number of people living with HIV, assessing treatment needs, and forecasting the HIV epidemic spread [4, 5].

The primary challenge in PSE arises from the hidden nature of KPs, often inaccessible through traditional methods. The small size of these groups (typically 1–2% of the general population) makes estimation based on general population surveys impractical. Wide-spread stigmatization of HIV-risk behaviors require specialized research methodologies for eliciting honest responses [6]. Global efforts to combat HIV epidemics have produced diverse approaches to estimate KP size, categorized into indirect (population surveys, network scale-up method (NSUM)) and direct methods (census, enumeration, capture-recapture, and multiplier methods).

NSUM, endorsed by UNAIDS and the World Health Organization [3], estimates KP size by surveying the general population and assessing the size and composition of the respondents’ social networks. Initially developed to estimate earthquake victims in Mexico City in 1986 [7], NSUM identifies the proportion of target groups in the social networks of respondents in representative general population surveys [8]. Estimates obtained through NSUM aligned with those from other studies using different methods [9–11]. Unlike with direct inquiries about risky behavior, respondents find it easier to answer questions about the presence of ‘people who use drugs by injecting’ among their acquaintances. While underestimation due to question sensitivity may be possible [12], NSUM is believed to contribute to reliable and valid PSE [13–15]. Additionally, NSUM requires fewer resources [16], enables simultaneous estimation for multiple groups, and is effective at both national and regional levels [17]. Another advantage is that KPs members are not required to disclose their affiliation, and direct contact with them is unnecessary [18].

In Ukraine, the HIV epidemic remains concentrated within KP, specifically among people who inject drugs (PWID), men who have sex with men (MSM), sex workers (SW), and transgender people (TP). According to the latest available integrated bio-behavioral studies (IBBS) data in 2020, HIV prevalence was 20.3% among PWID [19] and 2.0% among TP [20]. As of 2021, MSM showed HIV prevalence of 3.9% [21], and SW 2.6% [22]. ‘Bridge populations’ (BPs), closely linked to KP, can facilitate HIV spread to the general population. In Ukraine, BP such as sexual partners of PWID, clients of SW, and partners of MSM are officially recognized as HIV high-risk groups [23], which necessitates an understanding of the population sizes for planning and implementation of prevention programs.

In Ukraine, studies on estimating the KP and BP sizes have been conducted since the 2000s. Over this period, data on their size at national and regional levels have been collected, and various methods have been tested and compared to identify the most accurate ones. The results of previous estimations of KP and BP sizes in Ukraine, disaggregated by year, methods, and estimations, are presented Table 1.

**Table 1:** Results of previous KP and BP size estimation in Ukraine

Year	Definition	Behavior recency	Methods	National estimates	Uncertainty Intervals
**PWID, people who injected drugs**					
2002	No age limit, urban population	-	Multipliers, capture-recapture, expert survey	560,000 [24]	-
2005	Age 13+		Multipliers	425,000	325,000-425,000 [25]
2009	Age 16+	past 30 days	Multipliers	357,000	230,000-360,000 [26]
2009	Age 10+	past 12 months	NSUM	358,000	285,000-389,000 [26]
2009	Age 10–19		Multipliers, triangulation	50,000 [27]	-
2012	Age 14+	past 30 days	Multipliers	310,000	279,000-388,000 [28]
2015	Age 10–19		Multipliers, triangulation	21,700 [27]	-
2016	Age 14+	past 30 days	Multipliers, capture-recapture	346,900	265,200–475,700 [29]
2018	Age 10–19		Multipliers, triangulation	6,700 [27]	-
2019	Age 14+	past 30 days	Multipliers, capture-recapture, successive sampling	350,300^1^, incl. 317,000 in GCA^2^	265,700 − 474,900 [30]
**MSM, men who had anal or oral sex with men**					
2005	No age limit		Multipliers, population survey	300,000	177,000-430,000 [25]
2009	Age 15+		Population survey	154,000	95,000-213,000 [26]
2009	Age 10+	past 12 months	NSUM	32,000	23,000–39,000 [26]
2009	Age 10–19		Multipliers, triangulation	20,000 [27]	-
2012	Age 14+	past 6 months	Multipliers	175,000	200,100–249,000 [28]
2015	Age 10–19		Multipliers, triangulation	11,300 [27]	-
2016	Age 14+	past 6 months	Multipliers, capture-recapture	181,500	88,000-181,500 [29]
2015	Age 10–19		Multipliers, triangulation	21,300 [27]	-
2019	Age 14+	past 6 months	Multipliers, capture-recapture, and successive sampling	179,400^1^, incl. 161,200 in GCA^2^	126,600 − 240,700 [30]
**SW, men or women who provided sexual services**					
2001	Women, no age limit, urban population		Qualitative, triangulation	180,000 [31]	-
2005	Women aged 12+		Multipliers, proxy respondent	180,000	110,000-250,000 [25]
2009	Women aged 13+	past 30 days	Multipliers	-	45,000–74,000 [26]
2009	Women aged 10+	past 12 months	NSUM	81,000	65,000–93,000 [26]
2009	Men aged 10+	past 12 months	NSUM	3,700	2,800-5,200 [26]
2009	Men and women aged 10–19		Multipliers, triangulation	15,000 [27]	-
2012	Women aged 14+	past 6 months	Multipliers	80,000	54,000–85,800 [28]
2015	Men and women aged 10–19		Multipliers, triangulation	6,000 [27]	-
2016	Men and women aged 14+	past 6 months	Multipliers, capture-recapture	80,100	56,800 − 112,300 [29]
2018	Men and women aged 10–19		Multipliers, triangulation	5,000 [27]	-
2019	Men and women aged 14+	past 6 months	Multipliers, capture-recapture, and reverse tracking	86,600^1^, incl. 76,900 in GCA^2^	48,800 − 113,200 [30]
**TP, women or men whose gender identity and gender expression did not align with the sex assigned at birth (incl. without transgender transition)**					
2020	Age 14+		Multipliers, capture-recapture, wisdom of the crowds​ ​	8,200	3,400 − 14,000 [32]
**Sexual partners of PWID, men and women who had sex with PWID**					
2009	Age 10+		NSUM	32,000	27,000–39,000 [26]
2009	Age 10+	past 90 days	Multipliers	654,000 [26]	-
**Clients of female SW, men who used the sexual services of female SW**					
2009	Age 10+		NSUM	285,000	248,000-322,000 [26]
2009	No age limit	past 7 days	Multipliers	830,000 [26]	-
2009	Men who paid women for sex, no age limit	past 12 days	Population survey	220,000 [26]	-
**MSM who have female partners**,^**3**^**men who had sex with both men and women**					
2009	Age 10+	past 12 months	NSUM	6,200 [26]	-
2009	No age limit	past 6 months	Multipliers	36,000 [26]	-

^1^ This includes the entire territory of Ukraine, including donetsk, luhansk regions, and the autonomous republic of crimea not under control of the ukrainian government

^2^ GCA - Government-controlled areas

^3^ Used as an indirect estimate of the number of female sexual partners of MSM

In 2018, a decision was made that due to the limited resources, IBBS would no longer be conducted on the national scale as before but in a sample of cities [33]. This presents a methodological challenge for multiplier-based PSE because extrapolating city-level estimates to other areas introduces an additional level of uncertainty. In this context, NSUM is considered a viable alternative, enabling PSE in all regions at once. Additionally, NSUM addresses gaps in strategic HIV/AIDS information, especially concerning BPs, last estimated in 2009 [26].

Before the 2020 study, PSE for KPs in Ukraine using NSUM was conducted in 2009 by the Alliance for Public Health in partnership with the Kyiv International Institute of Sociology [16], and this experience became a reference for similar studies in Moldova [34], China [15], and Iran [35]. Ukrainian data were used in additional analyses exploring methods to overcome NSUM limitations [36, 37].

In this article, we present the results of NSUM study of KPs (PWID, MSM, SW, TP) and BPs (sexual partners of PWID, clients of SW, and partners of MSM) in Ukraine in 2020, highlighting challenges and approaches to mitigate NSUM methodological issues. As NSUM evolves, our findings contribute to its ongoing development, particularly in addressing respondent bias and exploring new advancements.

## Methods

### Research method

The study used a telephone survey via the computer-assisted telephone interview (CATI) method. The survey exclusively used mobile phone numbers, selected in two stages. In the first stage, an algorithm randomly generated seven digits of the number for each existing prefixes of mobile operators in Ukraine (random digital dialing– RDD), ensuring equal probability of being included for each resident. The second stage involved validating (activating) the generated numbers, with controlled distribution of prefixes based on previous studies to ensure equal chances of inclusion for subscribers of different mobile operators in the sample.

### Sample and study geography

The survey targeted the entire population of Ukraine aged 14 and older, possessing mobile phones. Besides age, the inclusion criteria included residency in the surveyed area for the past 6 months and oral informed consent. The target sample size of 10,000 respondents was determined using the State Statistics Committee of Ukraine (SSCU) data, considering population distribution across regions, age categories, and gender. To ensure representativeness nationally and within each administrative unit, the target sample size was set at 400 respondents for each of the 24 regions and Kyiv City.

### Questionnaire

Adapted from the original 2009 study [16], questionnaire covered socio-demographic characteristics, social network size, the number of people in the social network belonging to specific KPs and BPs. To estimate the size of different subgroups within KPs by sex/gender and age, the survey questionnaire incorporated inquiries about the total number of acquaintances within each KP (e.g. *‘Do you know people older than 10 years who injected drugs in the last 12 months? How many of them?’*). Subsequently, respondents were asked to specify the gender distribution among these acquaintances, as well as to identify people within KPs aged 10 to 14 years and those aged 15 to 17 years. This methodology facilitated the calculation of KP subgroups by gender and age (10–14 years, 15–17 years, older than 15, older than 18), thereby enabling the estimation of KP size based on sex/gender and age.

Respondents also rated the level of social respect towards specific KPs in the community (“*I will name representatives of various groups, and you will tell me how much respect they have in your city/village. Please rate the respect on a scale from 1 to 5, where 1 is very low, and 5 is very high*”).

Before the survey, the questionnaire underwent pilot testing in a separate sample (*N* = 51) meeting inclusion criteria. The questionnaire was prepared in Ukrainian and Russian, and the survey was conducted in the language chosen by the respondent. The study questionnaire is available in Additional file 1.

### Defining KPs for respondents

Defining KPs for respondents involved using terminology familiar and non-stigmatizing terminology to avoid sensitivity issues and insincere responses. Therefore, language that respondents could understand and that maintained neutrality was crucial in questionnaire development. For instance, ‘people who inject drugs’ was used for PWID, ‘men who have sex with men’ for MSM, ‘people who provide sexual services for payment’ for SW, and ‘individuals who have changed their gender’ for TP. Recency intervals for risky practices were set at 12 months for PWID, MSM, and SW, while questions regarding TP did not specify temporal ranges. In the study, we estimated the population sizes of KPs and BPs aged 10 and above.

### Determining the size of a social network

Before the survey, respondents received the following introduction: “*I will be asking you about all your acquaintances aged 10 and above who reside in Ukraine. ‘Acquaintances’ refer to all the people you know and who know you by appearance or name, with whom you can contact if necessary and with whom you have interacted personally, by phone, or via email within the last two years. These can be members of your family or other relatives, friends, neighbors, coworkers, or people you learn from. It may also include those with whom you do not have good relations or whom you consider your enemies*”.

The study employed two methods to estimate the size of the respondent’s social network: the known population method (KPM) and the summation method [38]. For the KPM, 23 groups were chosen, meeting the recommendation of utilizing a minimum of 20 known populations [13]. The choice of a larger number aimed to reduce the standard deviation for the average social network size [39]. Groups were selected based on specific characteristics that facilitate easy identification of acquaintances, ensuring ‘visibility’ to the respondents and promoting heterogeneity. Inclusion of only one type, such as age or the presence of certain diseases, can lead to barrier effect [40]. The size of these groups ranged from 0.1 to 4.0% of the total population, adhering to methodological criteria.

The estimated population size of known populations was calculated based on respondents’ answers about the number of acquaintances, and the results were compared with available official information. The Estimated/Real Ratio (E/R ratio) was calculated, aligning with the approach used in other studies, providing greater internal validity and predictive reliability than regression [14]. To ensure accuracy, we used two models: the initial model included all known populations (23 groups), while the final model included only 13 populations for which the E/R ratio was closest to 1 (ranging from 0.7 to 1.5). Maximum likelihood estimation was used to estimate network size using the formula [12]:

1 $$ {c}_{i}=t\times \frac{{\sum }_{j=1}^{L}{m}_{ij}}{{\sum }_{j=1}^{L}{e}_{j}}$$

Where *c*_*i*_ is the estimated personal network size of person *i*, *t* is the size of the general population, *m*_*ij*_ is the number acquaintances for each respondent *i* in a specific known population *j*, and *e*_*j*_ is the actual size of known population *j*.

For the summation method, respondents were asked to count the number of acquaintances in each of the six categories: family members and relatives, friends and companions, acquaintances currently studying together, сo-workers and colleagues, neighbors, and other acquaintances.

Both sizes of the social network were used to estimate the size of KPs and BPs.

### Data collection

The survey, conducted from July to September 2020, used specialized Ukrainian software, OCA–CATI [41], for questionnaire programming, data entry, telephone database management, and survey recording during the data quality control. Survey hours were set from 12:00 to 21:00 daily, including weekends, to mitigate potential issues like unreachable numbers, refusals, or respondent dissatisfaction due to inconvenient calling times. Up to 3 repeated attempts were made for unanswered calls.

### Data quality control

External and internal measures were used to ensure data quality. External control verified data consistency with statistical information on key socio-demographic characteristics (age, gender, urban or rural residence). Internally, random interview recordings were checked against entered responses in the dataset, with an average of 20% of interviews undergoing quality control checks.

### Processing, weighting, and data analysis

Data were weighted based on the individual sampling probability, considering city-level population size, gender, age (using the SSCU data), the number of mobile numbers per respondent, and home language. Language weighting relied on past nationwide surveys by the Kyiv International Institute of Sociology conducted through face-to-face interviews. Weighting based on the number of mobile numbers per respondent accounted for the higher likelihood of inclusion for those with multiple phone numbers. At each weighting stage, coefficients were calculated for the six indicators and multiplied step by step.

To estimate the size of KPs and BPs within respondent’s social network, the formula was used [12]:

2 $$ e=t\times \frac{{\sum }_{j=1}^{L}{m}_{ij}}{{\sum }_{i=1}^{N}{c}_{i}}$$

Where *e* is the estimated size of the hidden population of interest, *t* is the general population size, *m*_*ij*_ is the number of acquaintances for each respondent *i* in hidden population *j*, and *c*_*j*_ is the estimated personal network size of person *i*.

To address the response bias, corrections were made, considering the level of social respect for each KP and BP. Respondents reporting fewer acquaintances from a hidden population, as perceived respect decreased served, as a measure of response sincerity. Correction of respondents’ respect ratings assumed that the most unbiased estimates came from those who rated respect as ‘average’, providing a neutral response. Since only a small proportion indicated ‘high’ or ‘very high’ respect, they were grouped together with ‘average’. Correction weights were used for each KP and BP based on respect level [8, 16]:

3 $$ {W}_{i}=\frac{{M}_{i}}{{M}_{3}}$$

Where *W*_*i*_ is the weight of each hidden population based on the level of respect (*i* varies from 1 to 5, representing the respondents who gave a specific respect rating to a particular hidden population), *M*_*i*_ is the average number of hidden population in the network of all people with respect level *i* for this hidden population, and *M*_*3*_ is the average number of hidden population in the network of people with a medium level of respect.

Additionally, a visibility factor correction was applied to address the respondents’ lack of knowledge about specific behaviors of their acquaintances. The analysis assumed the correct PSE as the initial estimate plus members of the hidden population not included in acquaintances’ network due to disrespect and ignorance of their affiliation. In our study, visibility data were available only for MSM. The 2018 IBBS among MSM revealed that only 9% openly share their sexual orientation, with 63% selectively concealing it and 29% hiding it from everyone. Most feel comfortable discussing it with close friends, while some confide only in close family [42]. Considering the risk of outing, we assumed that a broader circle of acquaintances might be aware of MSM’s sexual behavior than those who have been voluntarily informed.

If we presume that all acquaintances are aware of the behavior of MSM who do not conceal it, approximately half (50%) of acquaintances are aware of the orientation of those who selectively conceal it, and none are assumed to be aware of the orientation of those who hide it from everyone. The number of respondents who know about the MSM status can be estimated using the formula [16]:

4 $$ {c}_{informed}={p}_{1}\times {c}_{all}+\frac{{p}_{2}\times {c}_{all}}{2}$$

Where *c*_*informed*_ is the average size of social networks that may be aware of belonging to a hidden population, *p*_*1*_ is the proportion of representatives of the hidden population who completely conceal their affiliation, *p*_*2*_ is the proportion of representatives of the hidden population who partially conceal their affiliation, and *c*_*all*_ is the estimated personal network size.

The visibility correction coefficient is calculated using the formula:

5 $$ k=\frac{1}{{p}_{informed}}$$

Where *k* is the proportion of representatives of the hidden population who conceal their affiliation and *p*_*informed*_ is the proportion of the social network that may be aware of the affiliation to the hidden population.

Data processing and analysis were conducted using Ukrainian software “ОСА” [41], IBM SPSS Statistics 22, and Microsoft Excel. Outlier checks were performed during data analysis to identify and address excessively large or small values, resulting in the removal of approximately 5% of extreme values from respondents’ answers about the number of acquaintances in various groups (trimmed mean).

## Results

The average interview duration was 24 min. Out of 50,239 contacted individuals, 39,120 refused to participate, resulting in a 20% response rate. Of the 11,029 participants who consented, 1,029 interviews were interrupted, while 10,000 were successfully completed. Interview interruptions were mainly due to respondents expressing anger and discomfort when confronted with sensitive questions. Comprehensive data on the total numbers generated, reached, and not reached are presented in Table 2.

**Table 2:** Survey recruitment and response rate

Call Outcome	N	%
**Total generated valid phone numbers**	**169,000**	**100.0**
**Contact with the respondent established**	**50,239**	**100.0**
Interview conducted	10,000	19.9
Interrupted interview	1,029	2.0
Refusal of the interview	39,210	78.0
**Contact with the respondent not established**	**89,353**	**100.0**
Number is inactive, no answer	43,493	48.7
Call declined	29,697	33.2
Voicemail activated	9,848	11.0
Line busy	6,315	7.1
**Excluded Numbers**	**29,408**	**100.0**
Number out of reach zone	12,136	41.3
Number not in use	9,962	33.9
Location or current activity makes it impossible to conduct an interview	2,378	8.1
Poor call quality	1,832	6.2
Respondent is under 14 years old	881	3.0
Physical or mental issues with the respondent	678	2.3
Business organization, government body, or another institution	620	2.1
Respondent resides in temporarily occupied territories by Russia	387	1.3
Respondent does not permanently reside in the survey city	199	0.7
Respondent resides abroad	152	0.5
Respondent refused to disclose the place of residence	123	0.4
Respondent does not understand Ukrainian or Russian	60	0.2

### Estimates of the social networks size

For the KPM, the mean maximum likelihood estimate of the respondents’ social network size in the initial model, covering 23 groups, was 206 (SD = 182). The range of network sizes varied from 0 (indicating that the respondent did not know anyone from the listed groups) to a maximum of 1,261. The trimmed mean (5%) was 189. The E/R ratio for the 23 groups was 46/1, signifying a fivefold overestimation of the known population size compared to official data. Accuracy of estimates varied across group: the model accurately estimated 10 groups (ranging from 0.7 to 1.5), significantly overestimating the size of nine groups, and underestimated the size of four populations.

In the subsequent analysis, groups with overestimated or underestimated estimates were excluded, resulting in the retention of 13 groups in the final stage where estimated and official numbers closely matched. These groups accounted for 0.37 of the general population. The social network size in this refined model averaged 239 people, with a trimmed mean (5%) of 213 people. Compared to the initial model, this iteration better predicted the size of the specified groups, with the E/R ratio at 1.1 and a correlation of *r* = 0.729. Despite the overestimation of the number of men and women aged 20–29, they were retained in the analysis to avoid limiting the list of known population to relatively small categories. A comparison of the estimated size of known populations with their numbers according to official statistics and the E/R ratio for the initial and final models is presented in Table 3.

**Table 3:** Estimated size of known populations, compared with official statistics

Group Name	Data Source	Official Size	Initial Model		Final Model	
			Estimated Size	Estimated/Real Ratio	Estimated Size	Estimated/Real Ratio
Men aged 20 to 29	SSCU	2,477,429	7,467,254	3.0	6,600,253	2.7
Men aged 15 to 17	SSCU	589,638	2,116,213	3.6		
Men aged 70 and older	SSCU	1,480,459	1,861,517	1.3	1,645,382	1.1
Women aged 20 to 29	SSCU	2,346,114	5,661,998	2.4	5,004,600	2.1
Women aged 15 to 17	SSCU	557,536	1,928,096	3.5		
Women aged 70 and older	SSCU	3,280,073	1,973,938	0.6	1,744,749	0.5
Children (aged 10–13, boys and girls)	SSCU	1,827,827	2,193,101	1.2	1,938,467	1.1
People who died in 2019	SSCU	581,114	598,463	1.0	528,977	0.9
Individuals with disabilities	SSCU	1,123,098	7,467,254	0.6		
Men named Pavel (aged 14 and older)	Responses to the questionnaire^1^	336,494	513,859	1.5	454,196	1.3
Women named Oksana (aged 14 and older)	Responses to the questionnaire	610,031	754,303	1.2	666,723	1.1
Individuals completing postgraduate or doctoral programs in the last 5 years (regardless of dissertation defense)	SSCU	32,728	267,506	8.2		
Judges	Higher Qualification Commission of Judges of Ukraine	5,306	123,004	23.2		
Men officially divorced in 2019	SSCU	138,005	94,412	0.7	83,450	0.6
Women who gave birth to a child in 2019	SSCU	310,605	309,925	1.0	273,941	0.9
Individuals who died from malignant neoplasms (cancer) in 2019	SSCU	77,481	196,006	2.5		
Individuals who had COVID-19 in the last 6 months	Ministry of Health of Ukraine	123,303	132,888	1.1	117,458	1.0
Physicians of any specialty	Center for Medical Statistics of the Ministry of Health of Ukraine	154,265	1,345,324	8.7		
Individuals who owned a motorcycle/scooter	SSCU	866,224	644,259	0.7	569,456	0.7
Children attending kindergartens or nurseries	SSCU	1,230,398	1,226,912	1.0	1,084,459	0.9
Individuals aged 14 and older not using the Internet (stationary and mobile)	Responses to the questionnaire	5,545,904	362,069	0.1		
Individuals who visited the United States in 2019	Responses to the questionnaire	348,911	120,533	0.3		
Individuals who died during the military conflict in eastern Ukraine in the last 5 years	Personnel Center of the Armed Forces of Ukraine	5,669	220,826	39.0		
**Total E/R ratio**			**4.6**		**1.1**	

^1^ The size of certain ‘visible’ populations, for which official statistical data were not available or were outdated, was determined by surveying respondents about their affiliation with these populations. The validity of this approach has been previously demonstrated through modelling [43].

SSCU, State Statistics Committee of Ukraine

Based on the results of the summation method, the average social network of respondents was 125 people (considering the trimmed mean at 5%). This encompasses a range of acquaintances, with 26 individuals identified as co-workers and colleagues, 22 as family members and other relatives, 21 as friends and companions, 18 as acquaintances currently studying together, 14 as neighbors, and 62 as other acquaintances. Overall, the total number of acquaintances across all categories amount to 125 people.

### Estimates of the populations sizes

At the first stage of the analysis, the estimated population size were as follow: for PWID, 81,896 using the KPM and 139,854 using the summation method; for MSM– 8,795 and 15,019; for SW– 13,438 and 22,947, and for TP– 1,814 and 3,098 respectively, as shown in Table 4.

**Table 4:** Estimated size of KPs and BPs, initial results

Studied group	Known Population Method		Summation Method	
	Estimation	CI (95%)^1^	Estimation	CI (95%)
**PWID**	**81,896**	**68,846–94,946**	**139,854**	**117,568–162,139**
Aged 10–14 years^2^	4,863	3,181–6,771	8,305	5,431–11,563
Aged 15–17 years	7,388	5,194–9,925	12,616	8,870–16,950
Aged 15 years and older	77,033	59,747–83,675	131,549	102,031–142,893
Aged 18 years and older	69,645	52,104–74,973	118,933	88,979–128,031
Females	16,609	14,707–18,010	28,363	25,116–30,757
Males	65,287	63,886–67,189	111,491	109,097–114,738
**Female SW**	**12,033**	**8,927–15,138**	**20,548**	**15,245–25,851**
Aged 10–14 years	1,554	851–2,313	2,654	1,452–3,949
Aged 15–17 years	1,453	877–2,081	2,481	1,497–3,553
Aged 15 years and older	10,479	7,129–13,251	17,894	12,174–22,628
Aged 18 years and older	9,026	5,780–11,669	15,413	9,871–19,928
**Male SW**	**1,405**	**913–1,896**	**2,399**	**1,559–3,238**
Aged 10–14 years	140	0–287	239	0–490
Aged 15–17 years	611	175–1,065	1,043	298–1,819
Aged 15 years and older	1,265	752–1,719	2,160	1,284–2,936
Aged 18 years and older	654	17–1,232	1,117	29–2,104
**MSM**	**8,795**	**6,351–11,238**	**15,019**	**10,846–19,192**
Aged 10–14 years	281	104–470	479	178–803
Aged 15–17 years	430	143–738	735	244–1,261
Aged 15 years and older	8,514	5,630–10,407	14,540	9,614–17,772
Aged 18 years and older	8,084	5,264–9,907	13,805	8,990–16,918
**TP**	**1,814**	**1,338–2,289**	**3,098**	**2,286–3,909**
Under the age of 18	332	131–544	567	224–929
Aged 18 years and older	1,482	913–1,660	2,531	1,559–2,834
Male-to-female	782	490–998	1,335	837–1,704
Female-to-male	1,032	691–1,274	1,763	1,179–2,176
**Sexual partners of PWID**	**26,554**	**21,549–31,559**	**45,346**	**36,799–53,893**
**Female clients of male SW**	**9,337**	**7,398–11,275**	**15,944**	**12,634–19,254**
**Male clients of female SW**	**62,913**	**52,155–73,670**	**107,436**	**89,065–125,807**
**MSM who have female partners** ^**3**^	**5,061**	**2,723–7,399**	**8,642**	**4,650–12,635**

^1^ Confidence intervals are calculated for a fixed social network size

^2^ The estimated size of sub-populations is calculated based on the percentage of acquaintances of a specific age/gender within the corresponding group

^3^ Used as an indirect estimate of the number of female sexual partners of MSM.

To mitigate the response bias, correction weights were calculated for each respondent category based on their level of respect for each KP and BP. For instance, respondents who rated respect for PWID as very low received a weight of 0.256762 (0.57/2.23), low– 0.067672 (0.15/2.23), and average or high/very high– 1 (2.23/2.23). Using these coefficients, the average number of acquaintances in hidden populations was computed, as presented in Table 5. Since responses about the level of respect for each hidden population only constitute a tenth of the sample, the ‘group coefficient’ was applied to calculate confidence intervals. This involves the ratio of the weighted estimate to the unweighted estimate (e.g., for the PWID, it was 173.348/81.896 = 2.1). The corrected PSEs were substantially higher than the initial ones, and detailed in Table 6.

**Table 5:** Estimated number of acquaintances by the level of respect

Studied group	Level of Social Respect (n - sample size)		
	Very low	Low	Medium or high / very high
PWID	0.57 (597)	0.15 (154)	2.23 (57)
Female SW	0.04 (417)	0.15 (173)	0.36 (106)
Male SW	0.00 (512)	0.04 (112)	0.05 (67)
MSM	0.01 (550)	0.23 (89)	0.24 (93)
TP	0.01 (441)	0.05 (128)	0.03 (117)
Sexual partners of PWID	0.21 (517)	0.20 (160)	0.22 (96)
Female clients of male SW	0.01 (411)	0.23 (158)	0.28 (121)
Male clients of female SW	0.10 (436)	0.78 (137)	0.86 (145)
MSM who have female partners	0.03 (473)	0.07 (124)	0.05 (131)

**Table 6:** Estimated size of KPs and BPs, corrected for social respect

Studied group	Known Population Method		Summation Method	
	Estimation	CI (95%)^1^	Estimation	CI (95%)
**PWID**	**173,248**	**145,641–200,855**	**295,857**	**248,713–343,001**
Aged 10–14 years^2^	10,287	6,729–14,324	17,569	11,489–24,461
Aged 15–17 years	15,629	10,988–20,996	26,689	18,764–35,857
Aged 15 years and older	162,961	126,393–177,011	278,288	215,843–302,285
Aged 18 years and older	147,331	110,224–158,603	251,599	188,232–270,845
Females	35,136	31,112–38,099	60,001	53,132–65,065
Males	138,112	135,149–142,136	235,856	230,791–242,724
**Female SW**	**39,483**	**29,294–49,672**	**67,425**	**50,024–84,826**
Aged 10–14 years	5,099	2,792–7,589	8,708	4,764–12,958
Aged 15–17 years	4,768	2,878–6,828	8,141	4,912–11,658
Aged 15 years and older	34,384	23,392–43,480	58,717	39,946–74,248
Aged 18 years and older	29,616	18,965–38,289	50,576	32,389–65,388
**Male SW**	**6,418**	**4,172–8,664**	**10,960**	**7,122–14,793**
Aged 10–14 years	640	0–1,311	1,092	0–2,238
Aged 15–17 years	2,791	799–4,865	4,764	1,361–8,309
Aged 15 years and older	5,778	3,435–7,852	9,868	5,865–13,412
Aged 18 years and older	2,987	78–5,628	5,104	132–9,611
**MSM**	**36,112**	**26,078–46,146**	**61,668**	**44,534–78,802**
Aged 10–14 years	1,154	427–1,930	1,967	731–3,297
Aged 15–17 years	1,766	587–3,030	3,018	1,002–5,178
Aged 15 years and older	34,958	23,117–42,731	59,701	39,475–72,971
Aged 18 years and older	33,193	21,614–40,678	56,683	36,913–69,465
**TP**	**5,834**	**4,305–7,363**	**9,963**	**7,352–12,571**
Under the age of 18	1,068	421–1,750	1,823	720–2,988
Aged 18 years and older	4,766	2,936–5,339	8,140	5,014–9,114
Male-to-female	2,515	1,576–3,210	4,293	2,692–5,480
Female-to-male	3,319	2,222–4,097	5,670	3,792–6,998
**Sexual partners of PWID**	**36,401**	**29,540–43,262**	**62,162**	**50,445–73,879**
**Female clients of male SW**	**41,793**	**33,114–50,468**	**71,370**	**56,553–86,187**
**Male clients of female SW**	**124,716**	**103,390–146,040**	**212,978**	**176,560–249,396**
**MSM who have female partners** ^**3**^	**8,021**	**4,316–11,726**	**13,697**	**7,370–20,026**

^1^ Confidence intervals are calculated for a fixed social network size

^2^ The estimated size of sub-populations is calculated based on the percentage of acquaintances of a specific age/gender within the corresponding group

^3^ Used as an indirect estimate of the number of female sexual partners of MSM.

The visibility factor was computed only for MSM, as data on the visibility of PWID, SWs, TP and BPs (sexual partners of PWID, clients of SW, and partners of MSM) were unavailable. Using the network size of acquaintances based on the KPM, the average network size of those potentially aware of the MSM status was calculated as: 0.09 × 213 + 0.63 × 213/2 ≈ 86 people (or 40.5% of the entire social network). Subsequently, considering 86 people as the size of the network informed about the MSM status and applying correction based on the level of respect, the estimated MSM population size was 89,165 people. Employing the network size method of summation, the average network size of individuals aware of the MSM status was calculated as: 0.09 × 125 + 0.63 × 125/2 ≈ 51 people (or 40.5% of the entire social network). Hence, the estimated MSM population size, adjusted for social respect and the visibility factor, was 152,267 people, as presented in Table 7.

**Table 7:** MSM population size estimates, corrected for social respect and visibility

Studied group	Known Population Method		Summation Method	
	Estimation	CI (95%)	Estimation	CI (95%)
MSM	89,165	6,439–113,941	152,267	109,960–194,573
Aged 10–14 years	2,849	1,054–4,765	4,857	1,805–8,141
Aged 15–17 years	4,360	1,449–7,481	7,452	2,474–12,785
Aged 15 years and older	86,316	57,089–105,509	147,410	97,469–180,175
Aged 18 years and older	81,958	53,368–100,440	139,958	91,143–171,159

## Discussion

In this study, we used NSUM to estimate the sizes of KPs (PWID, MSM, SW, and TG) and BPs (sexual partners of PWID, clients of SW, and partners of MSM) in Ukraine. These results represent the latest information on the size of these populations in the country, contributing valuable insights for the planning and monitoring of HIV services. Out study indicates that NSUM can produce reasonably accurate estimates, comparable to those obtained through other methods listed in Table 1. NSUM is feasible and cost-effective approach for obtaining nation-wide estimates of hidden populations, especially when resources for conducting IBBS and other direct-estimation studies are limited [33]. In Ukraine, NSUM provides additional advantages, particularly in the context of significant population migration resulting from Russia’s full-scale war against the country [44].

### Social networks size

Differences in social network size were observed between the KPM (213 people) and the summation method (125 people), which deviates from previous studies in the USA [38]. This variance could be attributed to the distinct focus of each method on different types of social networks. Currently, there have been no studies in Ukraine that could verify or refute either method. Reports from other countries indicate social network sizes ranging from 138 to 536 people [38, 45–47], with some exceeding ‘Dunbar’s number’ of 150 people [48], potentially influenced by the prevalence of the Internet and online social networks [49]. Despite research exploring the connection between social networks in the real and virtual worlds [50], the Internet could potentially contribute to the increase in size from 175 people in the 2009 NSUM study in Ukraine [16] to 213 people in 2020. In our study, the social network size for internet users was 255 people compared to 165 among non-users. We explain the difference in the obtained results using the two methods to their distinct focus on types of social networks. While the summation method concentrates on ‘close ties’ within specific categories [51], the KPM implies ‘weak ties’ [52], encompassing individuals with infrequent communication who remain in virtual social network contacts.

In our study, ‘acquaintances’ were defined as ‘people you know and who know you by sight or name, with whom you can contact when needed and have been in touch over the past two years, either in person, by phone, or by email’ [12]. This is a relatively broad definition, that could lead to an overestimation of the social network size, and an underestimation of the population size. Studies in Iran suggest that using the criterion of having at least one contact with ‘acquaintances’ may result in a social network size of 114 people [53], In contrast, the definition of acquaintances with a two-year time criterion and communication methods used in NSUM yielded a result of 308 people [54]. In a study in Singapore, ‘acquaintances’ were those with whom respondents communicated through text messages, phone calls, or in person, and who also engaged in communal eating over one year, showing a specific level of agreement [55]. An experiment in Rwanda, which determined social connections using both the traditional definition of acquaintances and the indicator of shared meals in the last 12 months, resulted in an average social network size of 251 and 108 people, respectively. Using shared meals as a criterion for defining ‘acquaintance’ produced more reliable results than the widely used two-year formulation [17]. Further NSUM studies may refine the ‘acquaintance’ definition and validate the network boundary, resulting in more accurate estimates. Comparative studies in the Ukrainian context are also necessary.

While estimates of KPs size based on the summation method are deemed more valid than those based on the KPM, aligning with previous studies and falling within the uncertainty intervals for PWID, MSM, SW (overall and for age groups over 15 years), and TP, as indicated in Table 1, we acknowledge possible imprecision in the summation method. The primary concern lies in the inability to verify if respondents’ reported number of acquaintances in different relationships corresponds to reality. Situations where a network member is counted more than once were also possible, such as counting someone both as a relative and a colleague [8]. Moreover, a smaller social network size might be obtained when using six groups of acquaintances compared to the initial version of this list, which distinguishes 16 categories [38]. These factors could lead to an underestimation of social network size and an overestimation of KPs compared to the KPM.

### NSUM limitations

Despite efforts to minimize methodological limitations, there are potential biases that should be acknowledged, prompting a more cautious interpretation and the exploration of new strategies to minimize their impact. Initial estimates of KPs and BPs seemed to be underestimated compared to other available data in Table 1. Biases in the estimates might arise from the barrier effect (people associating with similar individuals) [40], the transmission effect (lack of knowledge about distant acquaintances’ characteristics [56]), and the sincerity effect (hesitancy to include these groups in one’s close circle of acquaintances).

To mitigate the barrier effect, we used a nationally representative sample of Ukraine’s population aged 14 and above, aiming to minimize socio-demographic influences– a challenge faced by researchers in other countries [54, 55, 57]. However, we acknowledge that a representative sample with a large number of respondents remains susceptible to the transmission effect due to the ‘visibility’ of specific populations among the respondents’ acquaintances [58].

Our study confirms that the ‘visibility’ of populations (transmission effect) may be associated with the lack of clear characteristics allowing the correlation of acquaintances with specific groups and with the stigmatization of these characteristics [58]. For example, the estimates of some known populations, BPs, and specific KPs’ subpopulations might be influenced by the transmission effect.

The survey asked about all acquaintances with disabilities (a known population for estimating the social network size), regardless of severity, though it could be assumed that respondents were more aware of more severe disabilities that were visible [59]. Trying to account for that, we used statistical data on the number of individuals with disabilities in groups I-II (who usually have visible signs of limited functioning). This could lead to an underestimation of this group, as evident from Table 3. We also observed a discrepancy with the common belief that respondents tend to underestimate smaller populations [9, 60], which may also be explained by the transmission effect. For known populations, the transmission effect was accounted for by initially including a larger number of them in the model (23) and checking the E/R ratio for them, resulting in the final model with an average E/R ratio of 1.1. Our study supports the postulate that using all known populations is not ideal for such estimates [54].

The transmission effect likely hindered the performance of NSUM in estimating the size of the BPs in our study. Except for the outdated 2009 NSUM study, there are no other estimates of the size of sexual partners of PWID, clients of SW, and female partners of MSM in Ukraine are not available. As shown in Table 1, despite the increase in the NSUM estimates the number of KPs from 2009 to 2020, the BP estimates remain markedly lower than 2009 multiplier-based estimates. While NSUM has been used to estimate the size of SW clients [15], questions about acquaintances in BPs pose challenging for respondents. It requires not only knowledge of the behavioral practices of directly acquainted individuals but also awareness of the characteristics of their sexual partners, which may not be readily disclosed.

The inability of respondents to clearly link their acquaintances to the studied populations likely influenced the obtained estimates of minor PWID and SW in our study. While our results are comparable to previously estimated sizes of these KPs overall, caution is advised when interpreting the sizes of minor subpopulations, as they were twice as high as the data obtained using other methods in Ukraine (Table 1). The underestimation of minor subpopulations in our study may be explained by the fact that respondents could attribute teenage acquaintances who used non-injection drugs, common among Ukrainian teenagers [19, 61], to the underage PWID. Similarly, respondents might have included in the category of SW not only individuals directly providing sexual services but also those who lead an active sexual life [62], engaging in sexual activities with the expectation of receiving gifts or other resources in return, a practice known as ‘transaction sex’, observed in female teenagers [63, 64]. Given the absence of a ‘gold standard’ in PSE methodology and the potential impact of various factors on its validity, applying the correction coefficients and triangulating the results with other available data in the country is appropriate.

For estimating the size of hidden populations, considering the level of stigmatization is critically important as it can selectively limit knowledge about acquaintances belonging to KPs and BPs. In Ukraine, as in other countries, KPs often face stigma [65], leading them to conceal their practices even from family members. Therefore, we applied a visibility correction for MSM, yielding a more realistic estimate, as was previously observed in Ukraine and Iran [35]. Due to stigma, respondents might hesitate to acknowledge representatives of hidden populations in their social networks, resulting in the insincerity effect during the survey. This effect varies across groups, with more pronounced reluctance for male SW and MSM, and less for PWID. Regarding BPs, the average social respect level was approximately 1.5 on a 5-point scale for all four of them: 1.5 for sexual partners of PWID, 1.6 for female clients of male SW and MSM with female partners, and 1.7 for male clients of female SW. Therefore, in this study, as well as in 2009, an adjustment for social respect was made. However, it should not be universally applied in other countries without prior refinement and validation. For instance, applying the Ukrainian experience in Moldova revealed that respondents with more acquaintances from KPs were more likely to show low respect toward such individuals [34]. The application of the social respect factor correction in China did not yield positive results for SW in one of the study locations, characterized as a resort area. Individuals with a disrespectful attitude toward SW residing there had a higher likelihood of being acquainted with SW [15].

One of the main conclusions of our study is the significance of comprehensive consideration of the impact of stigma. The ‘key to success’ in its application is the adjustment of the initial data for both visibility and social respect levels. Relying on only one of these adjustments is insufficient, as demonstrated by our estimates of BPs sizes. Similar to a study in China [15], our results indicated an underestimation of the MSM size when applying only the social respect correction to minimize the insincerity effect. Even after using both correction coefficients, the size of the minor MSM in our study likely remains underestimated compared to the available 2018 data. This emphasizes the ongoing need for further development of NSUM and testing new techniques to overcome inherent limitations.

For example, a promising approach for assessing visibility levels is the ‘game of contacts’, employed in Brazil among KPs [66]. This method takes the form of a game using playing cards and a game board, facilitating the assessment of transmission rate and potential variations in the size of social networks among PWID and the general population. However, potential limitations of these approaches include the requirement for an additional population-specific survey, such as IBBS, to assess the rate of disclosure and the proportion of acquaintances aware of their affiliation [14]. In Ukraine, IBBS is conducted with large sample sizes (500 people in each city), allowing for the inclusion of hard-to-reach KPs’ subpopulations, and is conducted tri-annually, serving as additional source for integrating the visibility factor or the ‘game of contacts’ into the survey.

### Survey limitations

In addition to the NSUM methodological limitations, we acknowledge constraints associated with data collection method. Despite the key advantages of CATI, namely its safety in the context of the COVID-19 pandemic [67, 68], and presumed lower susceptibility to interviewer effects [69, 70], we acknowledge that conducting the survey via mobile phones might lead to the underrepresentation of population segments with very low incomes, as some individuals might be unable to afford a mobile phone or service charges [8]. While this could potentially influence the estimation of KPs, such as PWID and SW, for whom drug trafficking or providing sexual services is a primary income source [19, 22], our assumption is that the impact is minimal, as predominantly older individuals refrain from using mobile communication, and, on average, have fewer acquaintances, being less likely to be familiar with representatives of KPs and BPs.

The respondents could face challenges in accurately enumerating acquaintances within specific groups within the survey’s time constraints. The precision could further affected, as some responses were provided as interval estimates (e.g., 10–15 people), leading to the recording of rounded-up numbers. Additionally, we observed a tendency when respondents were more likely to report numbers ending in 0 or 5, particularly for counts exceeding 10, which was also seen in other studies [14]. Based on the time required to answer questions about the size of a social network, especially when using the KPM, Bernard et al. recommend incorporating questions about social network size into nationwide surveys [8], followed by collecting information about the presence of KPs and BPs representatives among acquaintances. This approach can potentially be implemented in Ukraine through regularly conducted omnibus survey.

It is also important to note the potential impact of respondent fatigue during lengthy phone surveys. Factors such as difficulty in understanding questions verbally [71] and decreased attention during extended interviews could contribute to measurement errors. Respondents in telephone surveys are more likely to express dissatisfaction with the survey’s length compared to face-to-face interviews, possibly due to social distance and altered dynamics [72]. Conducting surveys over the phone may also hinder respondents’ comprehension of information and increase fatigue due to high cognitive load [71]. In this study, questions about the number of acquaintances in different categories saw the highest percentage of ‘difficult to answer’, particularly regarding acquaintances in different age categories. For instance, 14.7% of respondents found it challenging to specify the number of male acquaintances aged 20 to 29 years, and 10.6% faced difficulty with acquaintances aged 15–17 years. This may suggest a reluctance on the part of respondents to engage with questions requiring attention and time. In future research, minimizing this limitation by revising the list of known populations, excluding those that did not yield valid results in the current study, is advisable.

## Conclusion

Our study in Ukraine provides crucial insights into the size of KPs (PWID, MSM, SW, and TP) and BPs (sexual partners of PWID, clients of SW, and partners of MSM) in 2020. NSUM proves to be a valuable tool for estimating the size of the former, providing reliable results aligned with previous studies, but may not be as suitable for estimating the size of the latter. Our findings contribute to the ongoing development of NSUM, emphasizing the need for continued exploration of aspects like respondent bias and visibility factor.

### Electronic supplementary material

Below is the link to the electronic supplementary material.

Supplementary Material 1

## Data Availability

According to the data sharing policy of the Alliance for Public Health, the datasets generated and/or analysed during the current study are not publicly available due to privacy or ethical restrictions. However, it can be provided upon a reasonable request, which should be directed to office@aph.org.ua.

## Abbreviations

BP: Bridge Population
CATI: Computer-Assisted Telephone Interview
E/R ratio: Estimated/Real Ratio
IBBS: Integrated Bio-Behavioral Studies
KP: Key Population
KPM: Known Population Method
MSM: Men Who Have Sex With Men
NSUM: Network Scale-Up Method
PSE: Population size estimation
PWID: People Who Inject Drugs
SSCU: State Statistics Committee of Ukraine
SW: Sex Workers
TP: Transgender People
