# Supplementary material for: Size estimation of key populations and ‘bridge populations’ based on the network scale-up method in Ukraine
Source: BMC Public Health. 2024 Apr 8;24:979. doi: 10.1186/s12889-024-18501-1 (PMC11000406; doi:10.1186/s12889-024-18501-1)
Supplement: Supplementary file 1 — Supplementary Material 1 [file 12889_2024_18501_MOESM1_ESM.pdf]

Questionnaire (NSUM study, 2020)

The International Charitable Foundation "Alliance for Public Health" is conducting a survey of the population of Ukraine aged 14 and above regarding their acquaintances, life experiences, and perceptions of other people's lives. The results of this study are confidential. We hope that the outcomes of this project will significantly impact the fight against the HIV epidemic in Ukraine.

You will be one of 10,000 survey participants, and the survey will be conducted in all regions of Ukraine. Your participation is voluntary, and you can stop at any time without any consequences. All your responses are completely anonymous, and the results will only be used in aggregated form.

Could you please dedicate about 15 minutes of your time to answer the survey questions?

1 Yes
2 No -> END OF SURVEY

In which language would you like to take the survey?
1 Ukrainian
2 Russian

Thank you for agreeing to participate! By providing your consent, you confirm that you are 14 years or older and agree to take part in this study. Do you agree?

1 I agree to participate and confirm that I am 14 years or older
2 I am under 14 years old -> END OF SURVEY
3 I do not agree to participate (Could you please explain why? It is essential for us) (WRITE DOWN ____)

I will be asking you about all your **acquaintances aged 10 and above who reside in Ukraine**. 'Acquaintances' refer to all the people you know and who know you by appearance or name, with whom you can contact if necessary and with whom you have interacted personally, by phone, or via email within **the last two years**. These can be members of your family or other relatives, friends, neighbors, coworkers, or people you learn from. It may also include those with whom you have not very good relations or whom you consider your enemies

I will not ask you about specific individuals or inquire about any personal information about your acquaintances; I don't even need to know their names. I am only interested in knowing the quantity of the individuals you are referring to.

1. **Survey ID:**
2. **Survey Date:**
3. **Your gender** (choose one):

1	Male
2	Female

4. **How old are you?** (open-ended question) ______________________________________
5. **Survey Region:**

1	Kyiv City	10	Kyiv Oblast	19	Ternopil Oblast
2	Vinnytsia Oblast	11	Kirovohrad Oblast	20	Kharkiv Oblast
3	Volyn Oblast	12	Luhansk Oblast	21	Kherson Oblast
4	Dnipropetrovsk Oblast	13	Lviv Oblast	22	Khmelnytskyi Oblast
5	Donetsk Oblast	14	Mykolaiv Oblast	23	Cherkasy Oblast
6	Zhytomyr Oblast	15	Odesa Oblast	24	Chernivtsi Oblast
7	Zakarpattia Oblast	16	Poltava Oblast	25	Chernihiv Oblast
8	Zaporizhzhia Oblast	17	Rivne Oblast		
9	Ivano-Frankivsk Oblast	18	Sumy Oblast		

6. **Name of the settlement:** ___________________________ or -> END OF SURVEY

7. **Have you been living continuously in this settlement for the last 6 months?**

1	Yes
2	No -> 1. END OF SURVEY

8. **Type of settlement:**

1	Very large city (over 500,000 inhabitants)
2	Large city (100-499,000)
3	Medium city (20-99,000)
4	Small city (up to 20,000 inhabitants) or urban-type settlement
5	Village

9. **Survey duration:**

1	Start time of the survey:	hours	minutes
2	End time of the survey:	hours	minutes

BLOCK A. SIZE ESTIMATION

INTERVIEWER, ASK THE FOLLOWING TWO QUESTIONS SEQUENTIALLY FOR EACH POPULATION.
9. How many individuals from the (name of the population) do you personally know who live in Ukraine? (open-ended question, COLUMN 1) – (98 – DIFFICULT TO ANSWER, 99 – REFUSE TO ANSWER, 0 - DON'T KNOW ANY)

If the response to Question 9 is 0, 98, 99 – SKIP THE CORRESPONDING STATEMENT IN QUESTION 10.

10. Does anyone among them live outside your region / Kyiv? IF YES: How many? (open-ended question, COLUMN 2) (1 – yes, they live (WRITE DOWN THE NUMBER), 2 – no, they do not live / none, 3 – yes, all of them, 98 – DIFFICULT TO ANSWER, 99 – REFUSE TO ANSWER)

#	Population Name	9. Total in Ukraine	10. Does anyone among them live outside your region / Kyiv? IF YES: How many?
1	How many men aged 20 to 29 do you know?		
2	How many men aged 15 to 17 do you know?		
3	What about men aged 70 and older? How many do you know?		
4	How many women aged 20 to 29 do you know?		
5	How many women aged 15 to 17 do you know?		
6	What about women aged 70 and older? How many do you know?		
7	How many children (aged 10-13, boys and girls) do you know?		
8	How many people who died in 2019 do you know among your acquaintances?		
9	How many individuals with disabilities do you know?		
10	How many men named Pavel aged 14 and older do you know?		
10a	FOR MEN ONLY: Is your name, by any chance, Pavel?	1-Yes 2-No	
11	How many women named Oksana aged 14 and older do you know?		
11a	FOR WOMEN ONLY: Is your name, by any chance, Oksana?	1-Yes 2-No	
12	How many individuals completing postgraduate or doctoral programs in the last 5 years do you know, regardless of dissertation defense?		
13	How many judges do you know among your acquaintances?		
14	How many men officially divorced in 2019 do you know?		
15	Do you know people older than 10 years who injected drugs in the last 12 months? How many of them? (SKIP QUESTIONS 15a-15d IF ‘0’)		
15a	How many of them are aged 10-14?		
15b	How many are aged 15 to 17?		
15c	How many of these individuals are women?		
15d	How many are men?		
16	Among your acquaintances, are there people whose partners inject drugs (i.e., individuals in marital or steady relationships with them)? How many of them?		
17	How many women who gave birth to a child in 2019 do you know?		
18	Do you know girls older than 10 years who provided sexual services for payment in the last 12 months? How many of them? (SKIP QUESTIONS 18a-18b IF ‘0’)		
18a	How many of them are aged 10-14?		
18b	How many are aged 15 to 17?		
19	Do you know men (boys) older than 10 years who provided sexual services for payment in the last 12 months? How many of them? (SKIP QUESTIONS 25-26 IF ‘0’)		
19a	How many of them are aged 10-14?		
19b	How many are aged 15 to 17?		
20	Among your acquaintances, are there women who use sexual services of men for payment? How many of them?		
21	What about men who use sexual services of women for payment? How many of them?		
22	And how many individuals who died from malignant neoplasms (cancer) in 2019 do you know?		
23	And how many among your acquaintances had pneumonia in the last 6 months?		
24	And how many among your acquaintances had COVID-19 in the last 6 months?		
25	How many physicians of any specialty do you know?		
26	How many among your acquaintances own a motorcycle/scooter?		

27	Do you know men older than 10 years who had sex with men in the last 12 months? How many of them do you know? (SKIP QUESTIONS 27a-27c IF ‘0’)		
27a	How many of them are aged 10-14?		
27b	How many are aged 15 to 17?		
27c	Are there among these men those who also have sex with women? If yes, how many of them?		
29	Do you know people living with HIV? If yes, how many of them do you know?		
30	Are there among your acquaintances those in whose families there are children attending kindergartens or nurseries? If yes, how many such children do you know?		
31	Do you know people older than 10 years who have changed their gender? How many of them do you know? (SKIP QUESTIONS 31a-31c IF ‘0’)		
31a	How many of them changed their gender from male to female?		
31b	And how many changed it the other way around – from female to male?		
31c	How many of them are aged 18 and older?		
32	How many individuals aged 14 and older not using the Internet (stationary and mobile) do you know?		
33	Do you know people who visited the United States in 2019?		
33a	Did you happen to visit the United States in 2019?	1-Yes 2-No	
34	How many people who moved from Luhansk and Donetsk regions to your settlement in the last 5 years do you know?		
34a	What about to other regions of Ukraine?		
34b	And to another country?		
35	How many people who moved from the Autonomous Republic of Crimea to your settlement in the last 5 years do you know?		
35a	What about to other regions of Ukraine?		
35b	And to another country?		
36	Are there among your acquaintances people who went to work abroad for a short period (up to 2-3 months) in 2019?		
37	Are there among your acquaintances people who went abroad in 2019, have permanent employment abroad, and do not plan to return to Ukraine?		
38	How many individuals who died during the military conflict in eastern Ukraine in the last 5 years do you know?		

11. Now let's talk more about these groups. I will name representatives of various groups, and you will tell me how much respect they have in your city/village. Please rate the respect on a scale from 1 to 5, where 1 is very low, and 5 is very high (one answer option for each line)

(DIVISION OF SAMPLE INTO 10 PARTS, 4 QUESTIONS IN EACH)

ROTATION 1

#	Population Name	Very low	Low	Medium	High	Very high	Difficult to answer / don’t know
1	Men aged 20 to 29	1	2	3	4	5	99
16	Women who use sexual services of men for payment	1	2	3	4	5	99
17	Men who use sexual services of women for payment	1	2	3	4	5	99

ROTATION 2

#	Population Name	Very low	Low	Medium	High	Very high	Difficult to answer / don’t know
2	Men aged 15 to 17	1	2	3	4	5	99
11	People who injected drugs in the last 12 months	1	2	3	4	5	99
12	People whose partners inject drugs	1	2	3	4	5	99

ROTATION 3

#	Population Name	Very low	Low	Medium	High	Very high	Difficult to answer / don’t know
3	Men aged 70 and older	1	2	3	4	5	99
13	Women who gave birth to a child	1	2	3	4	5	99
14	Women who provided sexual services for payment in the last 12 months	1	2	3	4	5	99
15	Men who provided sexual services for payment in the last 12 months	1	2	3	4	5	99

ROTATION 4

#	Population Name	Very low	Low	Medium	High	Very high	Difficult to answer / don't know
4	Women aged 20 to 29	1	2	3	4	5	99
22	Individuals who owned a motorcycle/scooter	1	2	3	4	5	99
23	Men who had sex with men in the last 12 months	1	2	3	4	5	99
24	Men who have sex with men and women	1	2	3	4	5	99

ROTATION 5

#	Population Name	Very low	Low	Medium	High	Very high	Difficult to answer / don't know
5	Women aged 15 to 17	1	2	3	4	5	99
19	Individuals with pneumonia	1	2	3	4	5	99
20	Individuals who had COVID-19	1	2	3	4	5	99
21	Physicians of any specialty	1	2	3	4	5	99

ROTATION 6

#	Population Name	Very low	Low	Medium	High	Very high	Difficult to answer / don't know
6	Women aged 70 and older	1	2	3	4	5	99
7	Individuals with disabilities	1	2	3	4	5	99
25	People living with HIV	1	2	3	4	5	99

ROTATION 7

#	Population Name	Very low	Low	Medium	High	Very high	Difficult to answer / don't know
29	People who moved from Luhansk and Donetsk regions to other regions of Ukraine in the last 5 years	1	2	3	4	5	99
30	People who moved from the Autonomous Republic of Crimea to another country in the last 5 years	1	2	3	4	5	99
31	People who moved from Luhansk and Donetsk regions to other regions of Ukraine in the last 5 years	1	2	3	4	5	99
32	People who moved from the Autonomous Republic of Crimea to another country in the last 5 years	1	2	3	4	5	99

ROTATION 8

#	Population Name	Very low	Low	Medium	High	Very high	Difficult to answer / don't know
8	Individuals completing postgraduate or doctoral programs in the last 5 years	1	2	3	4	5	99
28	Individuals who visited the United States in 2019	1	2	3	4	5	99
35	Individuals who died during the military conflict in eastern Ukraine in the last 5 years	1	2	3	4	5	99

ROTATION 9

#	Population Name	Very low	Low	Medium	High	Very high	Difficult to answer / don't know
9	Judges	1	2	3	4	5	99
33	People who went to work abroad for a short period (up to 2-3 months) in 2019	1	2	3	4	5	99
34	People who went abroad in 2019, have permanent employment abroad, and do not plan to return to Ukraine	1	2	3	4	5	99

ROTATION 10

#	Population Name	Very low	Low	Medium	High	Very high	Difficult to answer / don’t know
10	Men officially divorced	1	2	3	4	5	99
18	Individuals who died from malignant neoplasms (cancer) in 2019	1	2	3	4	5	99
26	People who have changed their gender	1	2	3	4	5	99
27	Individuals not using the Internet	1	2	3	4	5	99

12. Now I will ask you about the number of your acquaintances in general. Please tell me, how many do you have...? (one answer option for each line)

#	Category of acquaintances	Number
1	Family members and other relatives	
2	Friends and companions	
3	Acquaintances currently studying together	

#	Category of acquaintances	Number
6	Co-workers and colleagues	
7	Neighbors	
8	Other Acquaintances	

BLOCK B. SOCIO-DEMOGRAPHIC BLOCK

13. What is your education level? (choose one)

1	Elementary education (less than 7 grades)
2	Basic (incomplete) secondary education (9 full grades)
3	Complete secondary education (11 full grades)
4	Unfinished higher education (less than 4 courses)

5	Basic higher education and vocational education (university or college of I-II accreditation levels, technical school)
6	Complete higher education (bachelor, master, university or institute of III-IV accreditation levels)
7	Other (specify________________________)
99	Difficult to answer / don’t know

14. Please tell me, do you work or not? If yes, what is your profession? If not, what do you do? (open-ended question) ____

INTERVIEWER, RECORD THE RESPONDENT'S FULL ANSWER. CODE THEIR PROFESSION, OR IF THE RESPONDENT IS NOT WORKING, ASK AND CODE THEIR FIELD OF ACTIVITY.

1	Physician of any specialty
2	IT specialist
3	Military personnel
4	Judge
5	Other profession (specify__________)

6	Retiree
7	Student
8	Unemployed
8	Other (specify________________________)

14. Do you use the internet (fixed or mobile) at home? (choose one)

1	Yes
2	No

15. Think about your health in the last six months. Have you simultaneously experienced any of the following symptoms… (multiple answer options)

1	High temperature
2	Dry cough
3	Shortness of breath, difficulty breathing
98	No answer
99	Difficult to answer / don’t know

QUESTIONS 16-17 FOR WOMEN ONLY: **16. Have you personally ever provided sexual services for payment?**
1 Yes 2 No 98 - DIFFICULT TO ANSWER, 99 – REFUSE TO ANSWER

17. Have you personally ever used injectable drugs in your life?
1 Yes 2 No 98 - DIFFICULT TO ANSWER, 99 - REFUSE TO ANSWER

QUESTIONS 18-20 FOR MEN ONLY: **18. Have you personally ever used injectable drugs in your life?**
1 Yes 2 No 98 - DIFFICULT TO ANSWER, 99 - REFUSE TO ANSWER

19. Have you personally ever had sexual relations with men?
1 Yes 2 No 98 - DIFFICULT TO ANSWER, 99 - REFUSE TO ANSWER

20 Have you personally ever provided sexual services for payment?
1 Yes 2 No 98 - DIFFICULT TO ANSWER, 99 - REFUSE TO ANSWER

INTERVIEWER, CONCLUDE THE SURVEY AND THANK THE RESPONDENT FOR THEIR TIME.
